# Soluble Urokinase Plasminogen Activator Receptor (suPAR) Predicts 28-Day and 90-Day Mortality in Emergency Department Patients with Chest Pain, Dyspnoea, or Abdominal Pain

**DOI:** 10.3390/diagnostics15222851

**Published:** 2025-11-11

**Authors:** Francesco Gavelli, Francesca Maria Giolitti, Matteo Vidali, Marta Montersino, Matteo Bertoli, Luca Molinari, Marco Baldrighi, Michela Beltrame, Pier Paolo Sainaghi, Mattia Bellan, Filippo Patrucco, Gian Carlo Avanzi, Luigi Mario Castello

**Affiliations:** 1 Department of Translational Medicine, Università degli Studi del Piemonte Orientale, 28100 Novara, Italymatteo.bertoli1@gmail.com (M.B.); luca.molinari@med.uniupo.it (L.M.); pierpaolo.sainaghi@med.uniupo.it (P.P.S.);; 2 Emergency Medicine Unit, “Azienda Ospedaliero-Universitaria Maggiore della Carità”, 28100 Novara, Italy; 3 Clinical Pathology Unit, Fondazione IRCCS Ca’ Granda Ospedale Maggiore Policlinico, 20122 Milano, Italy; 4 Department of Internal Medicine, “Azienda Ospedaliero-Universitaria Maggiore della Carità”, 28100 Novara, Italy; 5 Respiratory Diseases Unit, “Azienda Ospedaliero-Universitaria Maggiore della Carità”, 28100 Novara, Italy; 6 Internal Medicine Unit, “Azienda Ospedaliero-Universitaria SS. Antonio e Biagio e Cesare Arrigo”, 15100 Alessandria, Italy

**Keywords:** early mortality, risk stratification, emergency medicine, acute conditions

## Abstract

**Background:** Early stratification of patients at emergency department (ED) admission is crucial. The soluble urokinase plasminogen activator receptor (suPAR) has emerged as a promising biomarker to identify the worsening of different clinical conditions. We aimed at evaluating whether baseline suPAR values predict 28-day and 90-day mortality in patients presenting to the ED with different conditions. **Methods:** In this prospective observational study, we enrolled patients with dyspnoea (D), chest pain (CP), and abdominal pain (AP). suPAR levels, together with clinical and laboratory data, were recorded at ED admission. The data collected included 28-day and 90-day mortality data, as well as 28-day and 90-day hospital readmission; and their correlation with suPAR values was assessed. **Results:** We enrolled 298 consecutive patients (CP 23.8%, D 31.9%, AP 44.3%). suPAR was significantly higher in patients with dyspnoea, compared to both patients with chest and abdominal pain (5.50 [3.50–8.60], 3.20 [2.30–4.10], 3.20 [2.33–4.48] ng/mL, respectively; *p* < 0.001). suPAR plasmatic levels were also higher in patients admitted to semi-intensive or intensive care units compared to other patients (4.10 [3.15–8.05] vs. 3.50 [2.55–5.50] ng/mL, respectively; *p* = 0.049). suPAR levels were significantly higher in patients dead at 28 days than in survivors (12.65 [9.83–18.53] vs. 3.60 [2.60–5.48] ng/mL, respectively; *p* < 0.001). Using the stepwise logistic regression analysis, only suPAR emerged as an independent predictor of 28-day mortality with an odds ratio of 1.31 (95% CI 1.10–1.56). **Conclusions:** Baseline suPAR levels are an independent predictor of mortality in ED patients with chest pain, dyspnoea, or abdominal pain.

## 1. Introduction

Emergency department (ED) visits often represent the first medical contact for most patients during the exacerbation of an acute medical condition. It is estimated that every year, around 140 million people visit the ED in the United States [1], for medical problems that range from non-acute and non-life-threatening conditions to the most severe emergencies. However, except for the extremes of these conditions, it is often difficult for the emergency physician to identify those patients that may have a negative outcome over the following days. For this reason, different methods for early patient stratification have been developed, that may include the use of scoring systems—such as the SOFA score, the MEWS or the NEWS scores—as well as the evaluation of serum biomarkers, such as C-reactive protein, procalcitonin, or lactate [2,3,4,5].

In recent years, the soluble urokinase plasminogen activator receptor (suPAR) has emerged as a promising biomarker for identifying conditions of chronic inflammation [6]. Specifically, suPAR is released after the cleavage of the activated immune cells such as monocytes, macrophages, and activated T-lymphocytes [7]. Thus, both chronic and acute inflammatory states show an increase in plasmatic levels of suPAR, regardless of the origin of inflammation.

Elevated levels of suPAR have been shown to be associated with the risk of worsening chronic kidney disease [8] as well as hepatic disease [9,10]. Also, elevated suPAR values are associated with an increase in mortality in patients with atrial fibrillation [11] and septic shock [12]. As evidence grows regarding its prognostic capacity across a wide range of specific clinical conditions [13], we aimed to evaluate whether suPAR could be useful in predicting mortality in a population of patients presenting to the ED for chest pain, dyspnoea, or abdominal pain.

## 2. Materials and Methods

### 2.1. Patients

This monocentric prospective observational study was conducted at the EDsof a university hospital. Consecutive patients were included if (i) they were ≥18 yo and (ii) they were admitted to the ED for chest pain, abdominal pain or dyspnoea. Patients were excluded if they (i) were pregnant, (ii) had sustained major trauma (defined as an injury severity score > 15), or (iii) declined to sign an informed consent. The study protocol was approved by the Local Ethical Committee (Comitato Etico Interaziendale AOU Maggiore della Carità di Novara, CE244/20).

### 2.2. Study Design

At ED admission, we collected demographic data, medical history (past and recent), and regular outpatient pharmacologic treatments prior to ED admission. Data regarding physical examination and vital signs, as well as radiological and laboratory results (including complete blood count, red cell distribution width, haemoglobin, lactate, and C-reactive protein) were also collected. A 3 mL venous blood sample was then collected in a lithium-heparin tube and then stored at −80 °C for suPAR measurement; such measurements were performed at the end of the enrolment period, so that clinicians were unaware of suPAR plasma concentration. Both diagnostic and therapeutic management of the patients were not influenced by the study protocol. The destination of the patient after the ED visit was also collected, as well in-hospital mortality. After one and three months, a telephone follow-up interview was performed for each patient in order to gather information regarding their health status, with particular attention to subsequent hospitalizations or death.

### 2.3. suPAR Measurement

The collected samples, approximately 3 mL of venous blood in a test tube containing lithium heparin, were centrifuged for 6 min at 1300 rpm. The plasma obtained was subsequently transferred to a vial, numbered with pseudonymized code to ensure anonymity and frozen for storage at −80 °C. At the time of the dosage, the samples were thawed, centrifuged, manually defibrinated and vortexed; this treatment served to eliminate any interference from fibrin. Plasma suPAR concentrations were measured using the suPARnostic^®^ TurbiLatex kit (ViroGates, Birkerød, Denmark) on the ADVIA 1800 Chemistry System analyzer (Siemens Healthineers, Munich, Germany). The assay is a turbidimetric immunoassay performed according to the manufacturer’s instructions. The total repeatability, or within-laboratory precision, declared by the manufacturer was 11.4% (low level: 3.7 µg/L), 5.8% (medium level: 6.9 µg/L), and 6.1% (high level: 9.9 µg/L), while the values verified in our laboratory (3 replicates × 5 days scheme) prior to implementation were 14.8%, 12.1%, and 6.6%, respectively.

Of note, the manufacturer provides data on lot-to-lot variability (≤10%) and method comparison (correlation coefficient r = 0.959, with an allowed bias of ≤15% at comparison against suPARnostic^®^ ELISA, ViroGates, Birkerød, Denmark). All measurements were performed centrally on the same analyser with internal quality controls included in each run. Therefore, inter-operator variability was minimised.

### 2.4. Endpoints

The primary endpoint was to evaluate the all-cause mortality at 28 days from ED admission, and its correlation with suPAR plasma concentrations. Secondary endpoints were correlations between suPAR levels at ED visit and (i) discharge at 24 h, (ii) admission to medical or critical care ward, (iii) readmission over the following 28 days, (iv) readmission or death over the following 90 days.

### 2.5. Statistical Analysis

#### 2.5.1. Sample Size

The sample size was calculated using the minimum events-per-variable (EPV) ≥ 10, due to the scarce amount of data on this population in the literature. For a model hypothesising three predictors (Ps) and an EPV of 10, with a mortality rate (f) of 0.10, the required sample size was calculated as N = 10 × P/f = 10 × 3/0.10 = 300 patients. This sample size was also expected to provide sufficient statistical power for secondary endpoints.

#### 2.5.2. Statistical Analysis

The normality of distributions was assessed using Q-Q plots and the Shapiro–Wilk test. Quantitative and qualitative variables are described using median and [interquartile range], and absolute and relative frequencies, respectively. Differences between two groups for quantitative variables were assessed using either the parametric Student’s *t*-test or the non-parametric Mann–Whitney test. In the case of comparisons involving more than two groups, either the parametric ANOVA or non-parametric Kruskal–Wallis test was used, with Bonferroni correction applied for multiple comparisons. For qualitative variables, Fisher’s exact was employed, when necessary. Correlation between variables was evaluated through the Spearman correlation coefficient. The diagnostic accuracy of suPAR as a marker of increased mortality at 4 weeks was evaluated using the Receiver Operating Characteristic (ROC) curve analysis and reported as the area under the curve (AUC) with its corresponding confidence interval. A potential cut-off point was estimated using the Youden method, which identifies the threshold value maximising a function of sensitivity and specificity (Youden Index = sensitivity + specificity − 1). Predictors of mortality at 4 weeks were assessed through univariate and multivariate stepwise logistic regression analysis. Statistical significance was set at a *p* value of 0.05. Statistical analysis was performed with MedCalc software v23.1.3 (Mariakerke, Belgium) and SPSS v29.0 software (IBM, New York, NY, USA).

## 3. Results

### 3.1. Patient Characteristics

**Figure 1:**
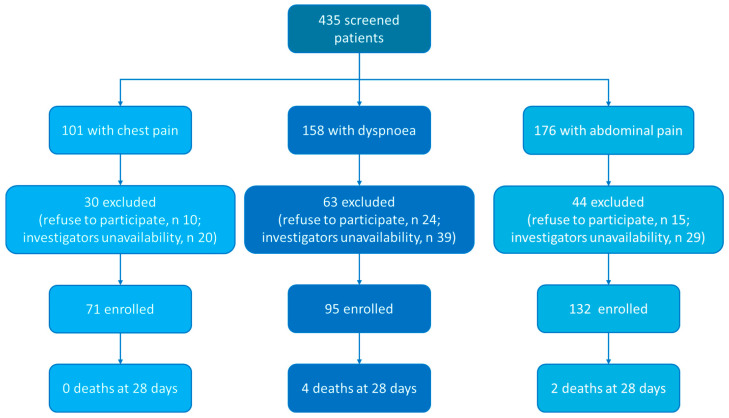
Study flow chart.

Between 1 February and 1 April 2021, we enrolled 298 patients (Figure 1). Fifty-five percent of them (*n* = 164) were male, and the median age was 59 [46–73] years. Seventy-one patients (23.8%) were admitted for chest pain, 95 for dyspnoea (31.9%) and 132 (44.3%) for abdominal pain. The Charlson comorbidity Index was 2 [0–4] points. At blood testing, haemoglobin was 14.0 [12.8–15.4] g/dL, C-reactive protein was 7.4 [1.3–53.0] mg/L and blood lactate was 1.0 [0.76–1.40] mmol/L. Most of the patients were discharged from ED (*n* = 170), 95 were admitted to a low-intensity ward and 33 were admitted to intensive or semi-intensive care unit. The remaining characteristics are reported in Table 1 and Table 2.

### 3.2. suPAR Levels at Baseline

**Figure 2:**
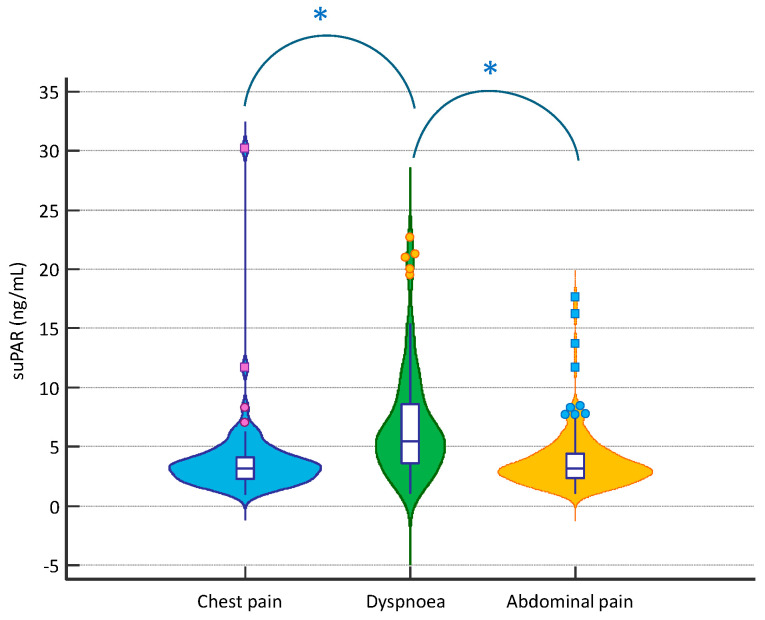
suPAR distribution according to the presenting symptom at the ED; *: *p* < 0.05.

In the overall population, baseline suPAR levels were 3.70 [2.60–5.55] ng/mL. No differences were observed in its values between men and women (3.80 [2.53–5.30] vs. 3.45 [2.60–6.15] ng/mL, respectively; *p* > 0.05). However, suPAR levels were significantly higher in patients with dyspnoea, compared to both patients with chest and abdominal pain (5.50 [3.50–8.60], 3.20 [2.30–4.10], 3.20 [2.33–4.48] ng/mL, respectively; *p* < 0.001 for comparison between dyspnoea and chest pain and dyspnoea and abdominal pain, *p* > 0.05 for comparison between chest pain and abdominal pain); in Figure 2, suPAR levels were significantly correlated with age (r = 0.495), with C-reactive protein levels (r = 0.627), creatinine (r = 0.381), and the Charlson comorbidity index (r = 0.474; *p* < 0.05 for all), but not with the body mass index (BMI) (r = 0.079; *p* > 0.05). In addition, plasma suPAR levels were higher in patients admitted to intensive and semi-intensive care unit compared to other patients (4.10 [3.15–8.05] vs. 3.50 [2.55–5.50] ng/mL, respectively; *p* = 0.049).

### 3.3. 28-Day Mortality

Six patients (2%) had died at 28 days (Table 1). suPAR levels were significantly higher in such patients than in survivors (12.65 [9.83–18.53] vs. 3.60 [2.60–5.48] ng/mL, respectively; *p* < 0.001). Non-survivors were also significantly older than survivors (83 [71–87] vs. 59 [45–72] years, *p* = 0.002). Also, blood lactate, the Charlson comorbidity index, C-reactive protein, red cells distribution width (RDW), creatinine, and haemoglobin were significantly different between patients dead at 4 weeks and survivors (Table 2). When all covariates that were statistically significant in the univariate analysis were entered into the stepwise logistic regression model, suPAR emerged as the only independent predictor of 28-day mortality, with an odds ratio of 1.31 (95% CI, 1.10–1.56; *p* = 0.0012).

**Figure 3:**
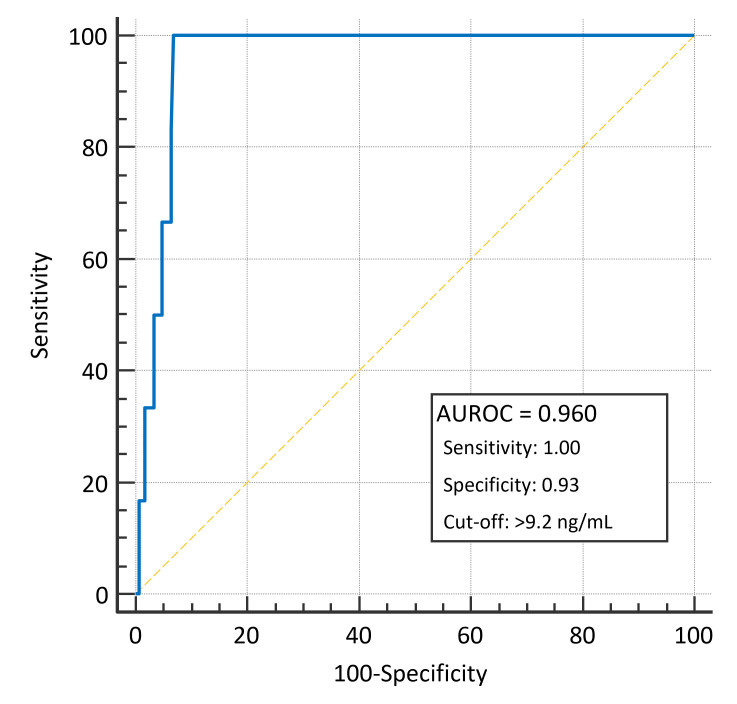
Area under the ROC curve for the ability of baseline suPAR plasmatic levels to predict 28-day mortality.

At the ROC curve analysis, baseline suPAR levels predicted for 28-day mortality with an AUROC of 0.960 (95% IC 0.932–0.983; *p* < 0.001) and with a sensitivity of 1.00 and a specificity of 0.93 with the optimal cut-off of 9.2 ng/mL (Figure 3). Specifically, in dyspnoeic patients, suPAR levels predicted 28-day mortality with an AUROC of 0.886 (95% IC 0.725–0.9011; *p* < 0.001) with a sensitivity of 1.00 and a specificity of 0.82 with the optimal cut-off of 9.2 ng/mL (Figure S1).

When dyspnoeic patients were divided according to the aetiology of their symptoms into cardiogenic (*n* = 30) and non-cardiogenic (*n* = 65), baseline suPAR values predicted mortality with similar accuracy among the two populations, with AUROCs of 0.875 (95% IC 0.703–0.967) and 0.905 (95% IC 0.806–0.964), respectively (*p* = 0.738 for AUROC comparison).

When we compared the diagnostic performance of suPAR with the one of C-reactive protein—which showed an AUROC of 0.835 (95% IC 0.717–0.954) (Figure S2)—we observed a significant difference among the two ROC curves (*p* = 0.0440).

### 3.4. Secondary Outcomes

Hospital readmission within 28 days was 6.7%, but baseline suPAR levels did not differ between these 20 patients and the other ones (4.20 [3.33–6.45] vs. 3.65 [2.60–5.50] ng/mL, respectively, *p* = 0.143). When 28-day mortality and 28-day hospital readmission were combined in a composite secondary outcome, suPAR was significantly higher in patients that reached such outcome compared to the other ones (6.10 [3.40–9.68] vs. 3.50 [2.53–5.30] ng/mL, respectively; *p* = 0.001).

Eight patients (2.6%) died at 90 days; baseline suPAR was significantly higher in these patients compared to the other ones (10.75 [8.85–16.63] vs. 3.55 [2.60–5.33] ng/mL, respectively; *p* < 0.001). Conversely, no differences were observed between the 46 patients (15.4%) admitted at 90 days and the other ones (3.90 [2.78–6.20] vs. 3.65 [2.60–5.50], respectively; *p* = 0.507). When 90-day mortality and 90-day hospital readmission were combined into a composite outcome, suPAR was significantly different between patients reaching such outcome and the other ones (4.55 [3.05–7.88] vs. 3.50 [2.53–5.28] ng/mL, respectively; *p* = 0.017).

At the ROC curve analysis, baseline suPAR plasmatic levels predicted 90-day mortality with an AUROC of 0.935 (95% IC 0.869–0.967; *p* < 0.001), with a sensitivity of 1.00, a specificity of 0.79, and a cut-off value of 6 ng/mL.

## 4. Discussion

The objective of this prospective, observational, single-centre study was to evaluate whether the plasma concentrations of soluble urokinase plasminogen activator receptor can serve as a predictor of mortality and as a useful tool to support ED physicians in the process of patient risk stratification and management. Physicians working in the ED are frequently faced with time-sensitive and challenging decisions, such as whether to discharge or admit a patient and, in the latter case, determining the most appropriate level of care. A biomarker that reliably predicts mortality, or conversely helps to identify patients at low risk, could substantially improve decision-making in the ED. The prognostic value of suPAR has already been investigated in recent years across a range of acute and chronic conditions, including respiratory tract infections, cardiovascular events, and diseases of gastroenterological, infectious, cardiological, and oncological origin [9,14,15,16,17,18,19].

In agreement with previous studies, suPAR levels were not influenced by sex and body mass index [20] but were found to be positively correlated with the Charlson Comorbidity Index [21]. Consistent with our results, non-survivors were significantly older than survivors, and suPAR levels were positively correlated with age, in line with previous evidence [21]. We also found a correlation with C-reactive protein, one of the most important biomarkers of inflammation.

The possible prognostic value of the biomarker within EDs has been previously studied in Denmark; a retrospective study demonstrated an association between high levels of suPAR and an increased risk of mortality and rehospitalization both at 30 and 90 days in a cohort of 4343 patients not selected based on diagnosis [22]. Another retrospective study including 17,312 patients demonstrated that the biomarker has a good ability to predict the risk of short-term mortality, especially in addition to the NEWS score [23]. Other studies have shown that adding the prognostic biomarker suPAR to triage systems in EDs could improve the prediction of short-term mortality and, on the other hand, may be a useful biomarker for the identification of patients that can be safely discharged, which will have a positive impact on unnecessary admissions of subjects at low risk of mortality or readmission [24,25].

The prognostic role of suPAR has also been analysed in the literature for specific pathologies related to the symptoms included in our analysis, such as acute coronary syndrome [26,27], pneumonia [14], sepsis [15], or acute pancreatitis [16]. However, no study has used a patient classification based on the main symptom of presentation. Our study focused on three symptoms that account for many ED presentations: chest pain, abdominal pain, or dyspnoea. While we did not demonstrate a potential role of suPAR in the diagnostic workup (only in patients with dyspnoea were suPAR levels significantly higher than in those with chest or abdominal pain), our data showed a significant association between high levels of suPAR and death within 28 days in the whole population of the study. Moreover, in the multivariate analysis, suPAR was the only independent predictor of mortality with an odds ratio of 1.31. Previously, a cut-off of 5.9 ng/mL had been set to identify patients at high risk of mortality [24]; the cut-off identified in our study was slightly higher (9.2 ng/mL). However, it should be noted that other studies have analysed mortality in different populations and that the mortality rate of our semi-undifferentiated ED population was relatively low (6%). Of note, suPAR diagnostic ability was superior to that of C-reactive protein.

We also analysed the impact of renal function, given that suPAR concentrations are known to be influenced by impaired clearance. Although creatinine correlated with suPAR at baseline and was associated with 28-day mortality in the univariate analysis, it did not remain significant in the multivariate model. This finding suggests that the prognostic value of suPAR is not fully explained by renal function and that suPAR may capture risk information beyond kidney dysfunction. Our data are consistent with previous reports indicating that suPAR reflects the systemic inflammatory and comorbidity burden, providing prognostic information independent of creatinine.

The role of suPAR as a predictor of mortality was also confirmed when considering a wider range of time: we found a statistically significant difference in baseline suPAR values comparing survivors and non-survivors at 90 days (3.55 and 10.75 ng/mL, respectively) and the ROC curve analysis showed that a baseline suPAR value of 6 ng/mL predicted 90 days mortality with AUROC of 0.935.

We also explored suPAR as a predictor of hospital readmission both at 28 and 90 days but the biomarker did not perform significantly in this regard. When mortality and readmission were combined in a composite outcome, suPAR levels were significantly higher in patients who experienced the combined outcomes both at 28 and 90 days.

Our findings that baseline suPAR concentrations independently predicted 28-day and 90-day mortality, regardless of presenting symptom or renal function, can be interpreted in light of the pathophysiology of suPAR release. The fact that suPAR originates from the cleavage of the membrane-bound urokinase plasminogen activator receptor on activated leucocytes reflects a sustained activation of the innate and adaptive immune systems rather than a transient acute-phase reaction. This persistent immune activation is a unifying mechanism across the different acute conditions included in our cohort. The fact that suPAR remained the only independent predictor of short-term mortality even after adjustment for creatinine underscores that it captures a global immune activation state that contributes to adverse outcomes across these heterogeneous clinical presentations. Of note, in our population, patients presenting with dyspnoea exhibited significantly higher baseline suPAR levels than those with chest or abdominal pain. This finding can be interpreted in light of the pathophysiological features of common causes of acute dyspnoea (e.g., heart failure, pneumonia, chronic obstructive pulmonary disease exacerbations) which are characterised by sustained activation of the innate and adaptive immune systems and by endothelial dysfunction.

Similarly, its superiority over C-reactive protein can be explained by the fact that the latter is an acute-phase protein synthesised in the liver with kinetics tightly governed by current IL-6/IL-1/TNF signalling; it excels at capturing transient inflammatory flares but may be less sensitive to the long-tail risk carried by chronic immune activation [28].

In practice, suPAR should be interpreted according to the context (including age, comorbidity, and renal function) to maximise its incremental value. Future implementation studies should test standardised “suPAR pathways” on patient flow, safety, and resource use.

In practical terms, suPAR may have potential as an adjunct to early warning scores (e.g., NEWS/MEWS) and routine biomarkers in the ED, as suggested by previous studies [23,24,25]. Although our data suggest that baseline suPAR independently predicts short-term mortality across different clinical presentations, further studies are needed to determine whether its use can improve risk stratification and patient management. The integration of suPAR into clinical decision-making tools should therefore be considered exploratory and requires validation in larger, prospective cohorts.

This study has some limitations. First, this is a single-centre study conducted in a university hospital, which may limit the generalisability of our results since patients’ management and clinical protocols can differ across institutions. Second, the number of enrolled patients was slightly lower than the one required by the sample size calculation, which could theoretically render our study underpowered. However, the number of patients was very close to the calculated requirement, so the impact of this limitation can be considered minimal. Third, our ROC analyses were performed on the whole cohort without an independent validation set; therefore, the identified cut-off values should be regarded as exploratory and require confirmation in external, larger populations. We also acknowledge that the low mortality rate observed in our cohort may have limited the statistical power of the multivariate analysis to detect independent predictors of mortality other than suPAR. For the same reason, the multivariable model should be considered statistically unstable and results should be interpreted with caution. Larger multicentre studies are, again, warranted to confirm our results. Moreover, although we collected a wide range of clinical and laboratory data, the possibility of unrecorded or residual confounding factors cannot be completely excluded.

## 5. Conclusions

Our findings support the role of suPAR as an independent predictor of short- and medium-term mortality in patients presenting to the ED with common acute symptoms such as dyspnoea, chest pain, and abdominal pain. suPAR may therefore represent a valuable tool to help ED physicians rapidly and easily identify high-risk patients who require greater resources, closer monitoring, more extensive diagnostic evaluation, and more aggressive treatment.

## Figures and Tables

**Table 1:** Baseline characteristics and laboratory findings.

Patients = 298	
Male sex (*n*, %)	164 (55%)
Age (years)	59 [46–73]
Body mass index	25.7 [23.1–28.4]
Peripheral oxygen saturation (%)	98 [95–99]
Charlson comorbidity index	2 [0–4]
Comorbidities (*n*, %)	
Arterial hypertension	139 (46.6%)
Chronic heart failure	11 (3.7%)
Atrial fibrillation/flutter	34 (11.4%)
Acute coronary syndrome	46 (15.4%)
Chronic obstructive pulmonary disease	8 (2.7%)
Stroke/transient ischemic attack	10 (3.3%)
Neoplasm	29 (9.7%)
Diabetes	34 (11.4%)
Presenting symptom (*n*, %)	
Chest pain	71 (23.8%)
Dyspnoea	95 (31.9%)
Abdominal pain	132 (44.3%)
Patients’ destination (*n*, %)	
Discharge	170 (57.0%)
Admission to low-intensity ward	95 (31.9%)
Admission to semi-intensive/intensive care unit	33 (11.1%)
28-day mortality (*n*, %)	6 (2%)
28-day hospital readmission (*n*, %)	20 (6.7%)
90-day mortality (*n*, %)	8 (2.7%)
90-day hospital readmission (*n*, %)	46 (15.4%)
White blood cells count (×10^3^/µL)	8.59 [6.50–10.93]
Haemoglobin (g/dL)	14.0 [12.8–15.4]
RDW (%)	13.3 [12.7–14.2]
Creatinine (mg/dL)	0.81 [0.64–1.02]
Blood glucose (mg/dL)	111 [97–131]
C-reactive protein (mg/L)	7.4 [1.3–53.0]
Lactate (mmol/L)	1.0 [0.76–1.40]
suPAR (ng/mL)	3.70 [2.60–5.55]

RDW: red cells distribution width. Data are presented as median and [interquartile range], and absolute and relative frequencies.

**Table 2:** Univariate analysis for 28-day mortality.

	Dead	Survived	*p* Value
Age (years)	83 [71–87]	59 [45–72]	0.002
Charlson comorbidity index	4.5 [3.8–5.3]	2.0 [0.0–4.0]	0.007
Blood lactate (mmol/L)	2.0 [1.2–5.6]	1.0 [0.7–1.4]	0.024
White blood cells (×10/µL)	10.06 [6.42–17.73]	8.57 [6.49–10.92]	0.325
Haemoglobin (g/dL)	10.7 [8.9–12.6]	14.1 [12.8–15.4]	0.001
RDW (%)	17.9 [13.5–21.6]	13.3 [12.7–14.2]	0.004
C-reactive protein (mg/L)	107 [21–219]	7 [1–47]	0.005
suPAR (ng/mL)	12.65 [9.83–18.53]	3.60 [2.60–5.48]	<0.001
Creatinine (mg/dL)	1.5 [1.0–2.0]	0.8 [0.6–1.0]	0.003

RDW: Red cell distribution width. Data are presented as median and [interquartile range].

## Data Availability

Individual, de-identified participant data are available from the corresponding author on reasonable request.

## Supplementary Materials

The following supporting information can be downloaded at https://www.mdpi.com/article/10.3390/diagnostics15222851/s1, Figure S1: area under the ROC curve for the ability of baseline suPAR plasmatic levels to predict 28-day mortality in dyspnoeic patients; Figure S2: area under the ROC curve for the ability of baseline C-reactive protein plasmatic levels to predict 28-day mortality in dyspnoeic patients.

## Abbreviations

The following abbreviations are used in this manuscript:

AUROC	area under the ROC curve
BMI	body mass index
ED	emergency department
RDW	red cells distribution width
ROC	receiver operating characteristic
suPAR	soluble urokinase plasminogen activator receptor

## References

[1] CDC—Centre for Disease Control, National Center for Health Statistics (U.S.) National Hospital Ambulatory Medical Care Survey: 2021 Emergency Department Summary Tables. https://www.cdc.gov/nchs/fastats/emergency-department.htm.

[2] Castello L.M., Gavelli F. (2024). Sepsis Scoring Systems: Mindful Use in Clinical Practice. Eur. J. Intern. Med..

[3] Guan G., Lee C.M.Y., Begg S., Crombie A., Mnatzaganian G. (2022). The Use of Early Warning System Scores in Prehospital and Emergency Department Settings to Predict Clinical Deterioration: A Systematic Review and Meta-Analysis. PLoS ONE.

[4] Usman O.A., Usman A.A., Ward M.A. (2019). Comparison of SIRS, qSOFA, and NEWS for the Early Identification of Sepsis in the Emergency Department. Am. J. Emerg. Med..

[5] Bruhn R., Skjøt-Arkil H., Skovsted T.A., Brasen C.L., Andersen E.S., Heltborg A., Hertz M.A., Petersen E.R.B., Mogensen C.B., Torres A. (2025). Biomarker Profiling for Infection Diagnosis in Emergency Departments: A Diagnostic Study Evaluating C-Reactive Protein, Procalcitonin, Club Cell Protein 16, Interleukin-6, Chitinase-like Protein, and Soluble Urokinase-Type Plasminogen Activator Receptor. Clin. Biochem..

[6] Rasmussen L.J.H., Petersen J.E.V., Eugen-Olsen J. (2021). Soluble Urokinase Plasminogen Activator Receptor (suPAR) as a Biomarker of Systemic Chronic Inflammation. Front. Immunol..

[7] Rehan S.T., Hussain H.U., Ali E., Kumar K.A., Tabassum S., Hasanain M., Shaikh A., Ali G., Yousaf Z., Asghar M.S. (2023). Role of Soluble Urokinase Type Plasminogen Activator Receptor (suPAR) in Predicting Mortality, Readmission, Length of Stay and Discharge in Emergency Patients: A Systematic Review and Meta Analysis. Medicine.

[8] Zhang Z., Liu C., Han Y., Li D., Huang Y., Shi K., Xia S., Wei J., Liu H., Sun L. (2025). The Predictive Value of suPAR for Glomerular Segmental Sclerosis Lesions in Renal Pathology. Ren. Fail..

[9] Artusa F., Lamatsch S., Phan M.D., Özdirik B., Berger H., Egerer M., Knorr-Klocke J., Fischer J., Veelken R., van Bömmel F. (2025). Soluble Urokinase Plasminogen Activator Receptor Predicts Survival and Hepatic Decompensation in Advanced Hepatocellular Carcinoma. Liver Int..

[10] Wang Y., Wu F., Chen C., Xu L., Lin W., Huang C., Yang Y., Wu S., Qi J., Cao H. (2021). Soluble Urokinase Plasminogen Activator Receptor Is Associated with Short-Term Mortality and Enhanced Reactive Oxygen Species Production in Acute-on-Chronic Liver Failure. BMC Gastroenterol..

[11] Wisborg F.D., El Caidi N.O., Taraldsen I.A., Tonning S., Kandiah A., El-Sheikh M., Bahrami H.S.Z., Andersen O., Rasmussen L.J.H., Hove J. (2025). Soluble Urokinase Plasminogen Activator Receptor (suPAR) as a Prognostic Biomarker in Acutely Admitted Patients with Atrial Fibrillation. J. Arrhythm..

[12] Turan C., Yurtseven A., Ozkaya P.Y., Azarsiz E., Saz E.U. (2024). The Role of Soluble Urokinase Plasminogen Activator Receptor (suPAR) as an Early Indicator of Mortality in Pediatric Septic Shock. J. Clin. Lab. Anal..

[13] Belvederi F., Leggeri S., Urbani A., Baroni S. (2025). suPAR as a Biomarker of Support in Different Clinical Settings. Clin. Chim. Acta.

[14] Luo Q., Ning P., Zheng Y., Shang Y., Zhou B., Gao Z. (2018). Serum suPAR and Syndecan-4 Levels Predict Severity of Community-Acquired Pneumonia: A Prospective, Multi-Centre Study. Crit. Care.

[15] Huang Q., Xiong H., Yan P., Shuai T., Liu J., Zhu L., Lu J., Yang K., Liu J. (2020). The Diagnostic and Prognostic Value of suPAR in Patients with Sepsis: A Systematic Review and Meta-Analysis. Shock.

[16] Aronen A., Aittoniemi J., Huttunen R., Nikkola A., Nikkola J., Limnell O., Nordback I., Sand J., Laukkarinen J. (2019). Plasma Level of Soluble Urokinase Plasminogen Activator Receptor (suPAR) Predicts Long-Term Mortality after First Acute Alcohol-Induced Pancreatitis. Eur. J. Intern. Med..

[17] Ohnewein B., Shomanova Z., Jirak P., Paar V., Topf A., Pylypenko L., Schäbinger M., Volg F., Hoppe U.C., Pistulli R. (2025). Dynamics of the Novel Cardiac Biomarkers sST2, H-FABP, GDF-15 and suPAR in HFrEF Patients Undergoing Heart Failure Therapy, a Pilot Study. J. Clin. Med..

[18] Bahrami H.S.Z., Jørgensen P.G., Hove J.D., Dixen U., Rasmussen L.J.H., Eugen-Olsen J., Rossing P., Jensen M.T. (2025). Soluble Urokinase Plasminogen Activator Receptor and Interleukin-6 Improves Prediction of All-Cause Mortality and Major Adverse Cardiovascular Events in Type 1 Diabetes. J. Intern. Med..

[19] Hessels L., Duijkers R., Schoorl M., Terpstra L., Thijs W., Boersma W. (2025). The Value of Soluble Urokinase Plasminogen Activator Receptor (suPAR) as Predictive Tool in Hospitalised Patients With Community-Acquired Pneumonia (CAP). Clin. Respir. J..

[20] Haupt T.H., Kallemose T., Ladelund S., Rasmussen L.J., Thorball C.W., Andersen O., Pisinger C., Eugen-Olsen J. (2014). Risk Factors Associated with Serum Levels of the Inflammatory Biomarker Soluble Urokinase Plasminogen Activator Receptor in a General Population. Biomark. Insights.

[21] Haupt T.H., Petersen J., Ellekilde G., Klausen H.H., Thorball C.W., Eugen-Olsen J., Andersen O. (2012). Plasma suPAR Levels Are Associated with Mortality, Admission Time, and Charlson Comorbidity Index in the Acutely Admitted Medical Patient: A Prospective Observational Study. Crit. Care.

[22] Rasmussen L.J.H., Ladelund S., Haupt T.H., Ellekilde G., Poulsen J.H., Iversen K., Eugen-Olsen J., Andersen O. (2016). Soluble Urokinase Plasminogen Activator Receptor (suPAR) in Acute Care: A Strong Marker of Disease Presence and Severity, Readmission and Mortality. A Retrospective Cohort Study. Emerg. Med. J..

[23] Rasmussen L.J.H., Ladelund S., Haupt T.H., Ellekilde G.E., Eugen-Olsen J., Andersen O. (2018). Combining National Early Warning Score With Soluble Urokinase Plasminogen Activator Receptor (suPAR) Improves Risk Prediction in Acute Medical Patients: A Registry-Based Cohort Study. Crit. Care Med..

[24] Schultz M., Rasmussen L.J.H., Kallemose T., Kjøller E., Lind M.N., Ravn L., Lange T., Køber L., Rasmussen L.S., Eugen-Olsen J. (2019). Availability of suPAR in Emergency Departments May Improve Risk Stratification: A Secondary Analysis of the TRIAGE III Trial. Scand. J. Trauma Resusc. Emerg. Med..

[25] Schultz M., Rasmussen L.J.H., Høi-Hansen T., Kjøller E., Jensen B.N., Lind M.N., Ravn L., Kallemose T., Lange T., Køber L. (2019). Early Discharge from the Emergency Department Based on Soluble Urokinase Plasminogen Activator Receptor (suPAR) Levels: A TRIAGE III Substudy. Dis. Markers.

[26] Lyngbæk S., Marott J.L., Møller D.V., Christiansen M., Iversen K.K., Clemmensen P.M., Eugen-Olsen J., Jeppesen J.L., Hansen P.R. (2012). Usefulness of Soluble Urokinase Plasminogen Activator Receptor to Predict Repeat Myocardial Infarction and Mortality in Patients with ST-Segment Elevation Myocardial Infarction Undergoing Primary Percutaneous Intervention. Am. J. Cardiol..

[27] Mehta A., Desai S.R., Ko Y.-A., Liu C., Dhindsa D.S., Nayak A., Hooda A., Martini M.A., Ejaz K., Sperling L.S. (2020). Sex Differences in Circulating Soluble Urokinase-Type Plasminogen Activator Receptor (suPAR) Levels and Adverse Outcomes in Coronary Artery Disease. J. Am. Heart Assoc..

[28] Shell A., Vize C., Gianaros P., Rasmussen L.J.H., Marsland A.L. (2025). Executive Function and Soluble Urokinase-Type Plasminogen Activator Receptor (suPAR): A Longitudinal Study of Midlife Adults. Brain Behav. Immun..

