# Supplementary material for: Soluble Urokinase Plasminogen Activator Receptor (suPAR) Predicts 28-Day and 90-Day Mortality in Emergency Department Patients with Chest Pain, Dyspnoea, or Abdominal Pain
Source: Diagnostics (Basel). 2025 Nov 11;15(22):2851. doi: 10.3390/diagnostics15222851 (PMC12650818; doi:10.3390/diagnostics15222851)
Supplement: Supplementary file 1 [file diagnostics-15-02851-s001.zip › diagnostics-3930060-supplementary.pdf]

Supplementary Materials

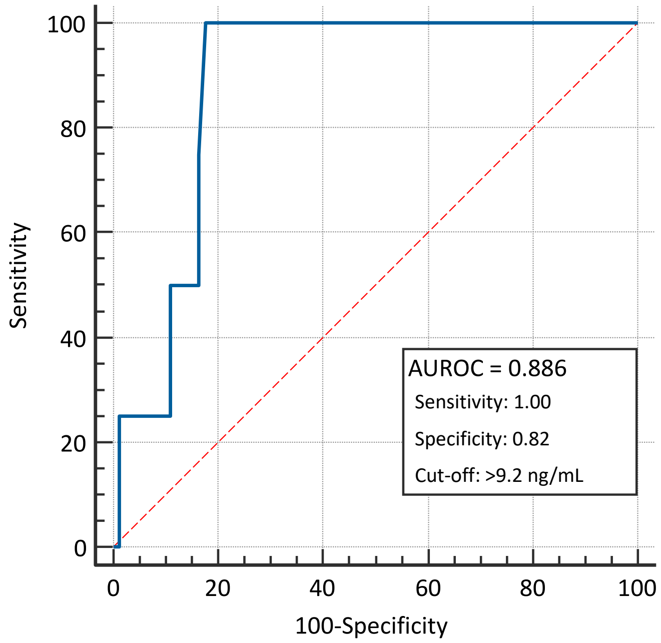

Figure S1. Area under the ROC curve for the ability of baseline suPAR plasmatic levels to predict 28-day mortality in dyspnoeic patients.

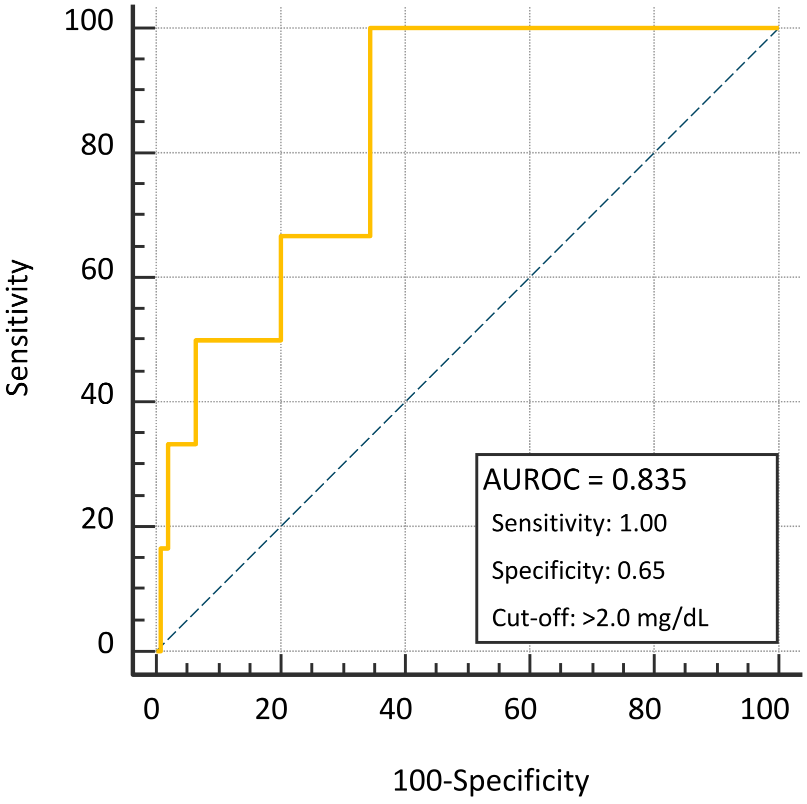

Figure S2. Area under the ROC curve for the ability of baseline C-reactive protein plasmatic levels to predict 28-day mortality in dyspnoeic patients.
